# Clinical therapeutic effects of probiotics in combination with antibiotics on periodontitis: A protocol for systematic review and meta-analysis

**DOI:** 10.1097/MD.0000000000023755

**Published:** 2021-01-29

**Authors:** Wenping Luo, Hao Li, Fei Ye

**Affiliations:** a Department of Stomatology, Wuchang Hospital of Wuhan; b Department of Stomatology, Wuchang Hospital Affiliated to Wuhan University of Science and Technology; c Department of Stomatology, Hankou Hospital of Wuhan, Wuhan, Hubei Province, China.

**Keywords:** antibiotic, efficiency, periodontitis, probiotic, systematic review

## Abstract

**Background:**

Dental pain can have a detrimental effect on quality of life. Symptomatic apical periodontitis is the most common cause of dental pain and arise from an inflamed or necrotic dental pulp. There is growing evidence to support the effectiveness of probiotics in combination with antibiotics on periodontitis. We therefor will conduct this study to evaluate the clinical therapeutic effects of probiotics in combination with antibiotics on periodontitis.

**Methods:**

We will systematically search the following databases: PubMed, the Cochrane Library, EMBASE, Web of Science, China National Knowledge Infrastructure (CNKI), Chinese BioMedical Literature Database (CBM), and WanFang database. A grey literature search will be conducted using ZETOC Conference Proceedings and Open Grey. Only randomized controlled trials (RCTs) related to research on probiotics in combination with antibiotics to treatment patients with periodontitis will be included. All sources have to be searched from their inception to October 2020. Two authors will independently select studies, extract study data, and evaluate the quality of the included studies. We will use Review Manager Software (RevMan 5.3) to analyze data.

**Results:**

This study will systematically evaluate the clinical therapeutic effects of probiotics in combination with antibiotics on periodontitis.

**Conclusions:**

This study will generate evidence for a better clinical decision of patients with periodontitis.

**Registration number:**

DOI 10.17605/OSF.IO/QZ6SB (https://osf.io/qz6sb/).

## 1 Introduction

Chronic periodontitis, which accounts for about 95% of patients with periodontitis, is an inflammatory disease of Asian tissues that uses bacteria as the motivating factor, which can activate the inflammatory immune response.^[1]^ As the disease progresses, it can lead to the destruction of periodontal supporting tissues, manifested as loss of periodontal attachment, destruction of alveolar bone, and eventually tooth loss.^[2]^ Conventional periodontitis treatment uses scaling and root planing (SRP) to remove microorganisms on the surface of teeth and the toxins they produce, thereby promoting the recovery of periodontal tissue.^[3–5]^ However, due to the local anatomical factors of some teeth and the immune defense of the host, SRP alone is difficult to eliminate pathogenic bacteria that invade the periodontal tissue, and its therapeutic effect is not ideal.^[6]^

Probiotics are a kind of beneficial active microorganisms that can prevent pathogen adhesion, inhibit bacterial growth, regulate mucosal immune system or cell proliferation, and promote the formation of new oral biofilms.^[7–9]^ In the medical field, probiotics have gradually been used to treat inflammatory bowel disease, respiratory infections, etc.^[10,11]^ In the field of oral cavity, studies have shown that probiotics have a potential therapeutic effect on oral pathogens, especially for children and adolescents dental caries.^[12]^ In recent years, studies have confirmed that probiotics play an important role in the prevention and treatment of periodontal diseases.^[13,14]^

## 2 Methods

### 2.1 Study registration

The present protocol has been registered in the Open Science Framework (OSF) (https://osf.io). Its registration DOI number was 10.17605/OSF.IO/QZ6SB. The statement of the Preferred Reporting Items for Systematic Reviews and Meta-Analyses Protocols (PRISMA-P) will be utilized as guidelines for reporting the present protocol.

### 2.2 Criteria for study selection

#### 2.2.1 Types of studies

Only randomized controlled trials (RCTs) related to evaluate the clinical therapeutic effects of probiotics in combination with antibiotics on periodontitis will be included.

#### 2.2.2 Types of participants

Participants who meet the diagnostic criteria of periodontitis will be included.

#### 2.2.3 Types of interventions

We will treat intervention group with any antibiotic (either intravenous injection or oral) at any dosage with probiotics.

#### 2.2.4 Types of outcomes

The primary outcomes will include visual analogue scale and clinician-reported measures of infection (e.g., swelling, temperature, trismus, regional lymphadenopathy or cellulitis). The secondary outcomes will include participant-reported quality of life measures and any adverse effects attributed to antibiotics.

### 2.3 Search strategy

We will systematically search the following databases: PubMed, the Cochrane Library, EMBASE, Web of Science, China National Knowledge Infrastructure (CNKI), Chinese BioMedical Literature Database (CBM), and WanFang database. A grey literature search will be conducted using ZETOC Conference Proceedings and Open Grey. Only randomized controlled trials (RCTs) related to research on probiotics in combination with antibiotics to treatment patients with periodontitis will be included. All sources have to be searched from their inception to October 2020. The search will be applied using the following combination of keywords and free word: periodontitis∗, probiotics∗, antibiotics∗, RCT∗, “randomized controlled trials”.

### 2.4 Data collection and analysis

#### 2.4.1 Selection of studies

**Figure 1:**
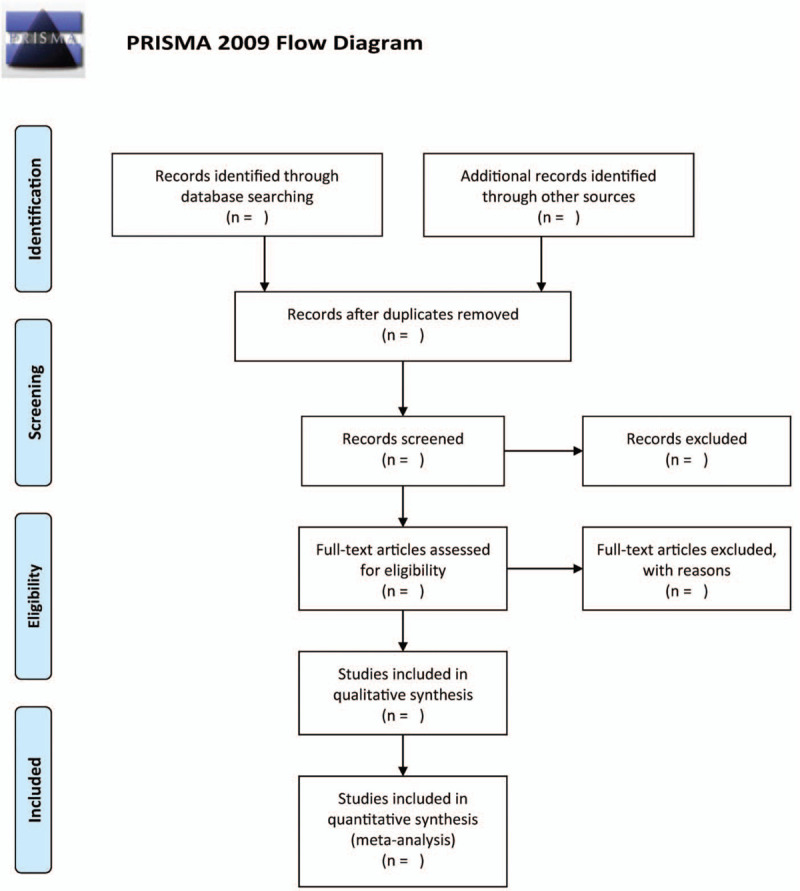
Flow diagram of the literature search.

EndNote X9 will be applied to manage literature. Two authors will independently screen the abstract/titles, and then review the full-text of the included studies. Any disagreement will be resolved by discussion or by consulting a third author where necessary. The flow chart is shown in Figure 1.

#### 2.4.2 Data extraction and management

Two authors will independently screen the literature and extracted data. The extracted data include: literature title, keywords, abstract, publication time, number of research cases, treatment plan and course of treatment, outcome indicator, etc. In the event of a dispute that cannot be resolved through discussion, a third author shall decide. If the original data cannot be described in detail, try to contact the author to supplement it, and then include it after completing the data.

#### 2.4.3 Assessment of risk of bias

Two authors will independently evaluate the risk of bias of the included studies based on the Cochrane Handbook. Any disagreement will be resolved by discussion or by consulting a third author where necessary.

#### 2.4.4 Measures of treatment effect

For dichotomous outcomes, we will plan to present the treatment effect as risk ratios (RR) together with 95% confidence intervals (CI). For continuous outcomes, we will plan to present the treatment effect as mean difference (MD) or standardized mean difference (SMD) and their corresponding 95% CI.

#### 2.4.5 Assessment of heterogeneity

We will plan to use the *I*^2^ statistic to evaluate heterogeneity among the included studies.^[15]^ If heterogeneity exist (*I*^2^ > 50%), we will plan to examine study reports to identify potential reasons for it, which will be estimated using the random-effects model.^[16]^

#### 2.4.6 Assessment of reporting biases

We will plan to create and examine a funnel plot to explore the potential publication bias of the included studies if we include more than 10 studies.

#### 2.4.7 Subgroup analysis

We will plan to undertake subgroup analysis based on possible identified reasons for heterogeneity if we identify sufficient studies.

#### 2.4.8 Sensitivity analysis

We will intend to repeat the analyses while excluding studies with high-risk or unclear methodological data if we identify sufficient studies.

### 2.5 Ethics and dissemination

The present study does not require ethical approval because no privacy patient data will be obtained.

## 3 Discussion

The prevention and treatment of periodontal disease is to control plaque and protect the periodontal ecosystem. Periodontal pathogens and their products are indispensable factors for inducing periodontitis.^[17,18]^ Effective removal of plaque biofilm is the primary goal of treatment of periodontitis. The use of SRP alone in the treatment of patients with chronic periodontitis has certain limitations. This study discusses that in non-surgical treatment methods, the adjuvant use of probiotics can compete with pathogenic bacteria for the combination of epithelial cells to form antibacterial compounds, and then combine antibiotic therapy to make treatment effect is better than pure SRP.^[9,13,17]^ To date, no prior systematic review has been performed to evaluate the clinical therapeutic effects of probiotics in combination with antibiotics on periodontitis. We therefore perform the present study and provide a comprehensive assessment of the clinical therapeutic effects of probiotics in combination with antibiotics on periodontitis. Our findings may provide generating reliable recommendations for the clinical management of periodontitis.

## Author contributions

**Conceptualization:** Wenping Luo.

**Data curation:** Wenping Luo, Fei Ye.

**Formal analysis:** Wenping Luo, Hao Li.

**Funding acquisition:** Hao Li, Fei Ye.

**Investigation:** Hao Li.

**Methodology:** Wenping Luo.

**Project administration:** Hao Li.

**Resources:** Hao Li.

**Software:** Wenping Luo.

**Supervision:** Hao Li, Fei Ye.

**Validation:** Wenping Luo.

**Visualization:** Hao Li.

**Writing – original draft:** Wenping Luo, Hao Li, Fei Ye.

**Writing – review & editing:** Wenping Luo, Fei Ye.

## Abbreviations

Abbreviations: CI = confidence intervals, MD = mean difference, RCT = randomized controlled trials, RR = risk ratios, SMD = standardized mean difference, SRP = scaling and root planning.
